# Mating and aggregative behaviors among basal hexapods in the Early Cretaceous

**DOI:** 10.1371/journal.pone.0191669

**Published:** 2018-02-21

**Authors:** Alba Sánchez-García, Enrique Peñalver, Xavier Delclòs, Michael S. Engel

**Affiliations:** 1 Departament de Dinàmica de la Terra i de l’Oceà and Institut de Recerca de la Biodiversitat (IRBio), Facultat de Ciències de la Terra, Universitat de Barcelona, Barcelona, Spain; 2 Museo Geominero, Instituto Geológico y Minero de España, Madrid, Spain; 3 Division of Entomology, Natural History Museum and Department of Ecology and Evolutionary Biology, University of Kansas, Lawrence, Kansas, United States of America; 4 Division of Invertebrate Zoology, American Museum of Natural History, New York, United States of America; Museum National d'Histoire Naturelle, FRANCE

## Abstract

Among the many challenges in paleobiology is the inference and reconstruction of behaviors that rarely, if ever, leave a physical trace on the environment that is suitable for fossilization. Of particular significance are those behaviors tied to mating and courtship, individual interactions critical for species integrity and continuance, as well as those for dispersal, permitting the taxon to expand its distribution as well as access new habitats in the face of local or long-term environmental change. In this context, two recently discovered fossils from the Early Cretaceous amber of Spain (ca. 105 mya) give a detailed view of otherwise fleeting ethologies in Collembola. These occurrences are phylogenetically spaced across the class, and from species representing the two major clades of springtails—Symphypleona and Entomobryomorpha. Specifically, we report unique evidence from a symphypleonan male (*Pseudosminthurides stoechus* Sánchez-García & Engel, 2016) with modified antennae that may have functioned as a clasping organ for securing females during mating on water’s surface, and from an aggregation of entomobryomorphan individuals (*Proisotoma communis* Sánchez-García & Engel, 2016) purportedly representing a swarming episode on the forest floor. We demonstrate that the mating behavioral repertoire in *P*. *stoechus*, which is associated with considerable morphological adaptations, likely implied elaborate courtship and maneuvering for guarantee sperm transfer in an epineustic species. These discoveries reveal significant behaviors consistent with modern counterparts and a generalized stasis for some ancient hexapod ethologies associated with complex mating and courtship and social or pre-social aggregations, so critical to specific constancy and dispersal.

## Introduction

Aside from trace fossils, some of the most remarkable examples of fossilized behaviors have been discovered in amber. Amber holds a special significance in paleobiological studies due to its ability to capture ‘snapshots’ of biotic interactions and behaviors directly, as well as to preserve organisms with sufficient life-like fidelity as to permit fine examination of morphologies directly linked to specific ecologies and ethological repertoires. Intraspecific interactions between individuals are critical to the survival of species, and yet these fleeting moments are virtually unknown for most extinct taxa [1]. Springtails exhibit a large suite of reproductive and developmental strategies [2–4]. They comprise a lineage of ubiquitous and ecologically varied hexapods, who together with the Protura and Diplura comprise the living sister group to the hyper-diverse insects [5]. Many species are common in soil and leaf litter where they influence on decomposition and nutrient availability, while others are arboreal and are abundant in rain forest canopies, and a relatively small number of species are water surface inhabitants [3, 6]. Springtails are also among the few arthropod groups that live successfully in polar regions, reflective of their considerable niche breadth.

**Fig 1:**
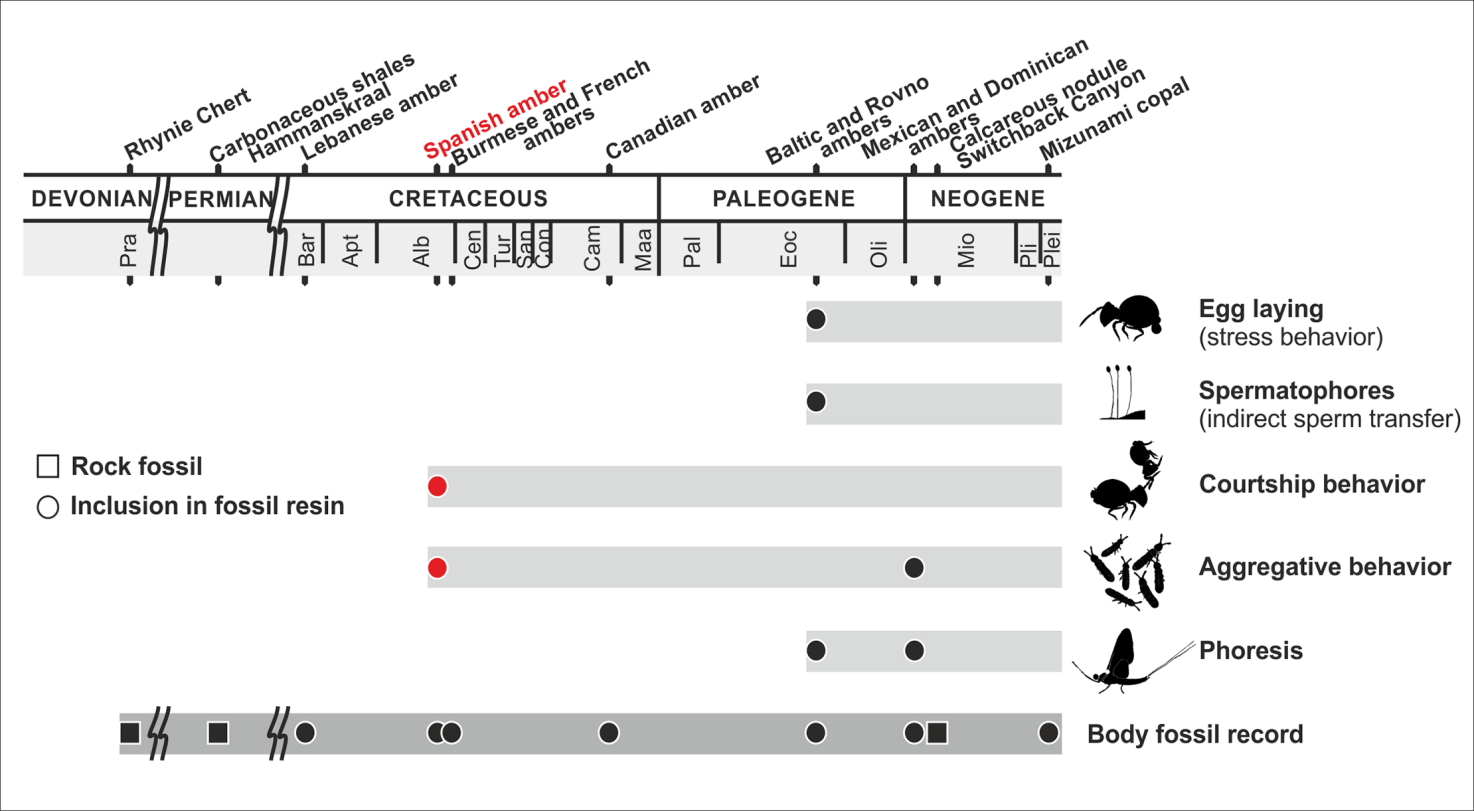
Fossil evidence of different springtails behaviors, and the geological range of the body fossil record. From top to bottom: *Sminthurus longicornis* depositing eggs (Baltic amber) [1]; *S*. *longicornis* with stalked spermatophores (Baltic amber) [12]; courtship behavior of *Pseudosminthurides stoechus* (herein described; Spanish amber); aggregation of *Proisotoma communis* (herein described; Spanish amber) and unidentified entomobryomorphans (Dominican amber) [13]; phoresy: *S*. *longicornis* over a harvestman, *Dicranopalpus ramiger* (Arachnida: Opiliones) (Baltic amber) [1], and unidentified sminthuridid on an oedemerid beetle [14], and a *Sphyrotheca*? sp. over a mayfly, *Borinquena parva* (Ephemeroptera) (Dominican amber) [15] (see also S1 Fig for a review of the general fossil record of Collembola).

Although the fossil record of Collembola extends back to the Early Devonian [7], representing some of the earliest evidence of Hexapoda [8, 9], the overall occurrence of the clade throughout this time period is scant prior to the Cretaceous. The bias of the fossil record of springtails towards preservation in amber is not surprising given their lightly sclerotized cuticle, minute size and water-repellant body structures which enhance buoyancy in water surface and prevent preservation as compression fossils (note that only a few collembolan records are in cherts, shale compressions, or calcareous nodules). Despite by the time of amber-producing deposits in the Early Cretaceous and onward the number of springtails preserved is higher [10, 11], albeit often of modest species diversity but of considerable phylogenetic breadth in representation, it remains that evidence for behavioral interactions is extremely rare. Rapid entombment and preservation in resin means that individuals can be preserved with life-like fidelity, sometimes even capturing behavioral interactions. The only previously described reproductive behaviors in fossil Collembola are those of individuals of *Sminthurus longicornis* in Eocene Baltic amber depositing eggs (most likely a stress response of interacting with the sticky resin) or with stalked spermatophores [1, 12], and a putative swarm of many individuals of an unidentified entomobryomorphan in Miocene Dominican amber [13]. These records are all of extant genera and none of the examples described are unusual by comparison to their living relatives, but they are helpful for understanding the general antiquity of these conditions. Other unusual cases of fossil behaviors and associated morphologies concern phoresy and include five *S*. *longicornis* aligned in a row and possibly attached by hooking their antennae over the leg of a harvestman, *Dicranopalpus ramiger* (Arachnida: Opiliones) in Baltic amber [1], and an individual of *Sphyrotheca*? sp. attached by its antennae over the basal anterior edge of the right forewing of the mayfly, *Borinquena parva* (Ephemeroptera) in Dominican amber [14] (Fig 1 and S1 Fig).

Here we report evidence for intraspecific interactions from two different species of springtails in Early Cretaceous Spanish amber—*Pseudosminthurides stoechus* and *Proisotoma communis—*which also happen to be the earliest hitherto known records of their particular behaviors: the presence of male clasping organs for courtship and securing the female during copulation, and an aggregation (a form of social or pre-social interaction in Collembola). In addition, they also shed new light on the edaphic and aquatic habitats of the Cretaceous forest.

## Materials and methods

### Materials and deposits

The Spanish amber pieces described here (accession numbers MCNA 11231 and MCNA 12788) are deposited in the Museo de Ciencias Naturales de Álava, Vitoria-Gasteiz, Spain (MCNA).

Amber pieces were prepared by trimming, grinding, and embedding in EPO-TEK 301 synthetic resin under vacuum for optimal viewing and curation. Reshaping of piece MCNA 11231 was made between a compromise to access further taxonomic details of the specimens and to preserve the syninclusions in their relative positions. The inclusions were examined by using transmitted and reflected light through compound microscopes Motic BA310 and Olympus BX41. We also use here the ultraviolet light as a tool to analyze the results of taphonomical processes in the amber pieces.

Spanish amber harbors a diverse biota from the Cretaceous, and to date, 16 different hexapod orders have been reported [16]. Amber is principally found in localities distributed in an arc from the east to the north along the Iberian Plate, which corresponds approximately to the seashore during the Early Cretaceous. Two main amber-bearing outcrops are found in the eastern area of the Basque-Cantabrian Basin (BCB) in the north Iberian Plate: Moraza, also named Peñacerrada I (in Burgos Province) which has yielded the two amber pieces under study, and Peñacerrada II in Álava Province. The amber from both outcrops belongs to the so-called ‘Álava amber’. Both amber deposits were included in the upper part of the Escucha Formation ([16] and references therein), which was defined from the Maestrazgo Basin (MB) (Easter of the Iberian Plate), as the result of a progradation–retrogradation–progradation process of a deltaic-swamp system [17, 18]. After the genetic interpretation proposed by [19] the upper member of the Escucha Fm. was considered as genetically related with levels from the Utrillas Fm. (located above) and no with their own two inferior members. This new lithostratigraphical redistribution was informally defined as Utrillas Group [19]. The upper member of the Escucha Fm. and the Utrillas Fm. (i.e. Utrillas Group) belong to a regional desert erg-system resulted from the aridification of the area. The limit between the Escucha Fm. and the Utrillas Group was described as a regional angular unconformity associated with a syn-sedimentary extensional tectonics, erosion and sedimentary bypass. This discontinuity separates the development of gymnosperm dominated forests under tropical-intertropical climate that promoted lignite deposits, Lower Albian in age (Coal bearing system [19]), from the development of an expansive desert during the Lower Albian since the Lower Cenomanian (Desert system [20, 21]). In the southern margin of the BCB, the Escucha Fm. and the Utrillas Group occurs, and the discontinuity is identified by [22]. These lithostratigraphic units mainly comprised of siliciclastic deposits that accumulated in continental to shallow marine environments [23, 24]. The Peñacerrada I amber outcrop is included in the clay-silt rich in organic matter levels from the bottom of the Utrillas Group (corresponding to the upper levels of the classical Escucha Fm.). These amber-bearing levels have been recently dated by palynological assemblages as Late Albian in age [22], and correspond to the fore-erg area *sensu* [25] between the erg system and the Proto-Atlantic Sea (coastal erg-margin systems) where swamps and marshes that originated clay deposits rich in organic matter developed, and where resins deposited. The Peñacerrada I outcrop is divided into three stratigraphic intervals [23]: the lower interval represented by interbedded mudstones, fine-to coarse-grained sandstones, and coal seams with bivalve and coal fragments; the middle interval by tabular strata of coarse-grained sandstones with carbonaceous fragments and macrofloral remains with encrusted surfaces towards the top; and the upper interval by fine-grained and well-sorted sandstones, non-cemented, covered by a heterolithic interval of mudstones with macro-floral remains, and sandstones. Amber is mainly found in the coal and fine-sand rich in organic matter levels.

### Imaging

Photomicrography was performed with a Moticam 2500 digital camera attached to the Motic BA310 compound microscope with Motic Images Plus 2.0 software. General photograph of piece MCNA 11231 was taken using Zeiss SteREO Discovery V12 stereomicroscope. Drawing was made using a drawing tube Olympus SZX-DA attached to an Olympus SZX9 stereomicroscope. To better illustrate the three-dimensional inclusions, photomicrographs were combined by using the software package Helicon Focus.

## Results

### Courtship behavior

**Fig 2:**
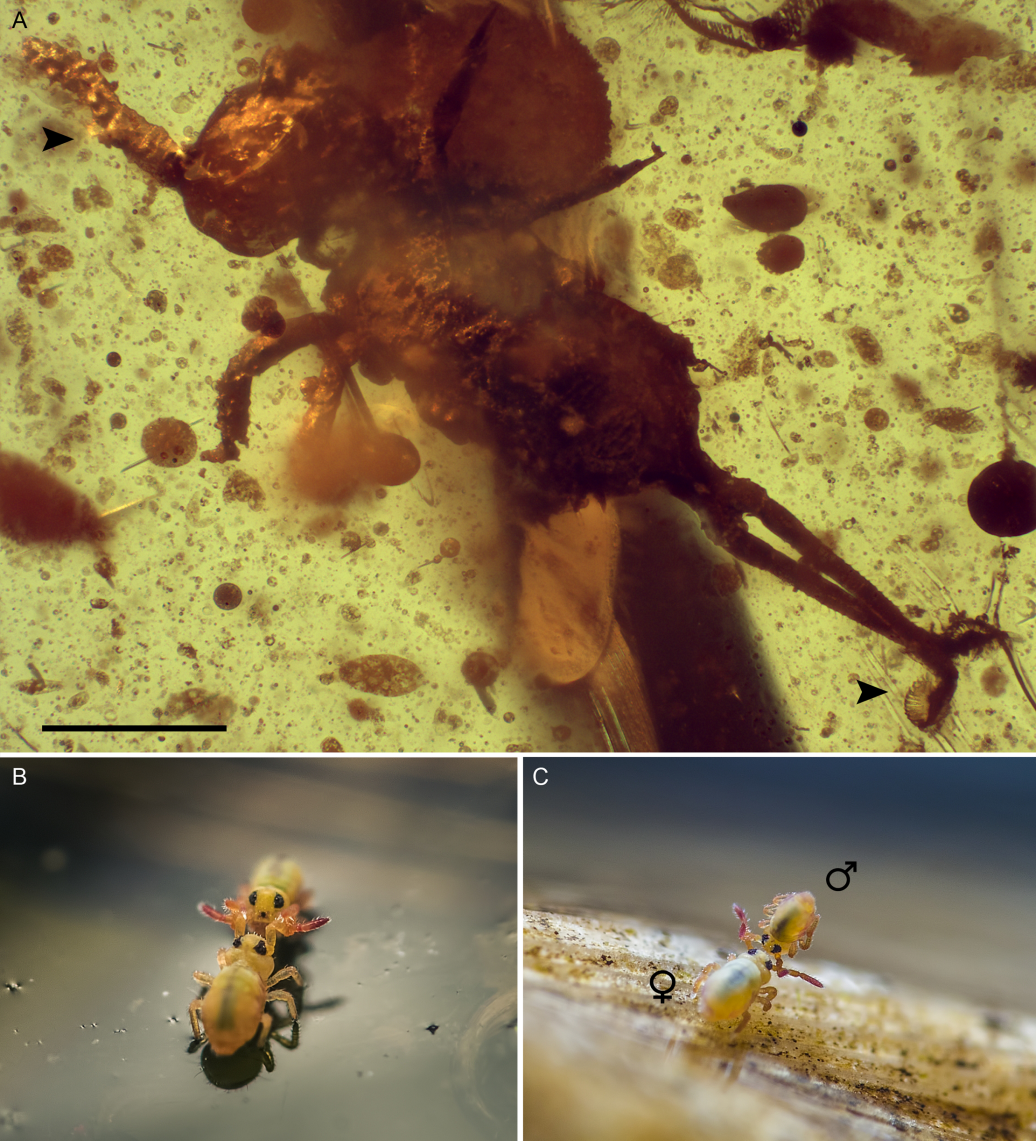
Fossil and extant Sminthurididae showing mating behavior and/or its underlying anatomy. (A) Dorsal habitus of the male of *Pseudosminthurides stoechus* (holotype; accession number MCNA 12788) as preserved in Early Cretaceous Spanish amber. Left arrow indicates the left modified clasping antenna. Right arrow indicates the wing-like enlarged mucrones, an adaptation to the epineustic way of life. (B and C) Reproduction biology of Recent *Sminthurides aquaticus* in a pond (coordinates: 52°16'11.9"N 8°06'46.4"E; Osnabrück, Germany) (images credit K. Beck, with permission). Pair formation on the water surface (B). The female carries the smaller male raised in the air as part of the courtship behavior (C). Body size of *S*. *aquaticus* is up to 1.0 mm in females, and 0.3–0.4 mm in males. Scale bar, 200 μm (A).

In Collembola, fertilization is through the transfer of spermatophore, which occurs in a variety of fashions, and sometimes implies dimorphic structures correlated with a number of behavioral attributes [3]. In the order Symphypleona there are taxa which exhibit obvious morphological differences between males and females [26]. One is a peculiar form of the male antennae, and which is particularly conspicuous in the family Sminthurididae [26–29]. Such specializations are present in the newly discovered sminthuridid *Pseudosminthurides stoechus* from Albian-aged amber from the Peñacerrada I outcrop, which is represented by a well-preserved individual positively identified as a male (S1 Text). Extant members of this family (145 species in 10 genera) [30] are all small, less than 1 mm in length, and are distributed throughout the world and often found on the surface of water. In the males, the antennae are modified for clasping the female during mating and the females lack anal appendages [31]. Among sminthuridids, *P*. *stoechus* has a unique combination of traits including a body size about 0.6 mm, a pair of large interocular vesicles, a distinctly tuberculate tibiotarsus, a head and abdomen lacking spines and broadened setae, the ‘ABC’ bothriotrichia distributed in an oblique line, and characteristic dens and mucro [10]. The antenna of male *P*. *stoechus* shows a developed first antennomere, and is modified in the second antennomere to interlock with the third and form a ‘clasping organ’ (Fig 2A). Among the diversity of modified antennae observed in extant sminthuridid genera, they mainly vary in the number, position, and morphology of the elements in the second and third antennomeres, usually formed of distinct papillae and accompanied by several trichobothria and other setae [31]. The morphology of the antennal elements in *P*. *stoechus* represents yet another morphotype within the overall syndrome observed in modern sminthuridids to serve this clasping function.

As in the primitively wingless insects, adult molting in Collembola imposes certain necessities on the reproduction of such species, which cannot retain stored sperm through ecdysis. In those species in which there are multiple reproductive instars (i.e., molting continues beyond sexual maturity), a female must collect a new spermatophore in order to lay fertile eggs, as sperm received during a prior, sexually mature instar is no longer retained. Although not surprising given the comparatively young age, Eocene stalked spermatophores do provide direct evidence of indirect sperm transfer at that time [12]. From this evidence it was hypothesized that the development of stalks in the springtail spermatophore may have evolved in groups which were specialized to live on the soil surface. In extant springtails, stalked spermatophores play a critical defensive role against predators, and appear to have evolved in response to pressures to isolate the sperm droplets from direct contact with antagonistic organisms inhabiting the soil and litter [3]. In other species the males deposit their sperm droplets directly on the substrate, and some exhibit direct transfer of sperm to the female’s genital aperture, albeit not through copulation via an intromittent organ as in most insects [3]. Courtship behaviors can also be diverse, ranging from complete absence to sophisticated ‘dances’ and tactile interactions. Formation of mating pairs has mainly been observed in the Symphypleona, and elaborate activities are used to entice a female toward the nuptial structure for pick up and fertilization [3, 32]. These habits are more ritualized in the Sminthurididae, where males perform an elaborate courtship and maneuver to aid with fertilization, accompanied by species-specific clasping modifications of the antennae [33]. Understandably, sperm transfer is hampered on the water surface. Spermatophores deposited directly on this medium are vulnerable, and direct sperm transfer and morphological modifications to facilitate transfer are common in aquatic species [29], thereby raising the success of fertilization.

The discovery of a Mesozoic sminthuridid male with modified antennae sheds new light on the early evolution of mating behaviors in Collembola. In *Pseudosminthurides stoechus*, the modified antennae with morphology similar to extant sminthuridids demonstrates that the clasping function was already developed at that time, and, most importantly, the associated behaviors of grasping the female during mating had already appeared and become specialized. Moreover, the morphology of the furca (the characteristic ‘spring’ of the abdomen), with the mucrones differentiated to form wing-like structures and the dentes bearing long and straight setae, along with the tuberculate tibiotarsus and the elongate unguis (claws), imply that *P*. *stoechus* likely had a epineustic lifestyle. Collembola have unwettable cuticles, and Recent aquatic species use their unguis and ventral tube (the only wettable areas) as a mechanism of anchorage to the water's surface [34]. Elongate claws such us that of *P*. *stoechus* have been reported in hydrophilous springtails, especially among neustic species, and facilitate movement on the water surface [6, 35]. Also, surface roughness is related to wettability [34], and the presence of macrotubercles on the tibiotarsus of *P*. *stoechus* may have enhanced its hydrophobic properties. Similar to its extant relatives, the elongated claws in *P*. *stoechus* may have broken through the water surface film, giving purchase and traction, whereas the unwettable nature of the tibiotarsus and the enlarged mucro and setae, may have provided the buoyancy necessary to allow the animal to move on the water’s surface without penetrating the surface tension. Accordingly to the specialized morphologies in the Spanish fossil, there must have already been in place a repertoire of stereotyped movements for mating, particularly involving elaborate courtship as evidenced by the clasping antennae, which collectively ensured the appropriate species response and/or positioning for sperm uptake on the water surface. Most of the cases of strong sexual dimorphism are related to aquatic habitats, and where males have clasping antennae to grasp females in order to avoid losing them through movement of currents [29]. The behavior observed for mating pairs of extant relative *S*. *aquaticus* (Fig 2B and 2C) on the water’s surface, often involves dance-like movements which are believed to facilitate the finding and uptake of the spermatophores. Males use their antennae to interlock with those of the females, and are carried around by their partners (Fig 2C), which activates them to deposit spermatophores and guide the females over the deposition site to enable the uptake of sperm [36]. The Early Cretaceous *P*. *stoechus* represents the first documentation of such significant ecobehavioral anatomical specializations within the clade, which likely responded to similar selective pressures as are known from extant counterparts in Recent ecosystems, and provides evidence of an early mating specialization, behavior, and aquatic life, similar to the biology of its modern relative, *S*. *aquaticus*. Analogous behaviors are observed among the basal orders of insects, and maybe represent a groundplan feature of Hexapoda, prior to the eventual origin of copulation and intromittent organs.

### Aggregative behavior

Certain species of springtails aggregate at times, and in some cases gather together in masses and may also migrate considerable distances over the soil surface (i.e., swarming aggregations) [37–44]. Some dense aggregations have been counted to comprise hundreds of millions of individuals [39]. Preservation of fossil soil communities is rare, and the only previously described record of a collembolan aggregation is from Miocene Dominican amber [13].

**Fig 3:**
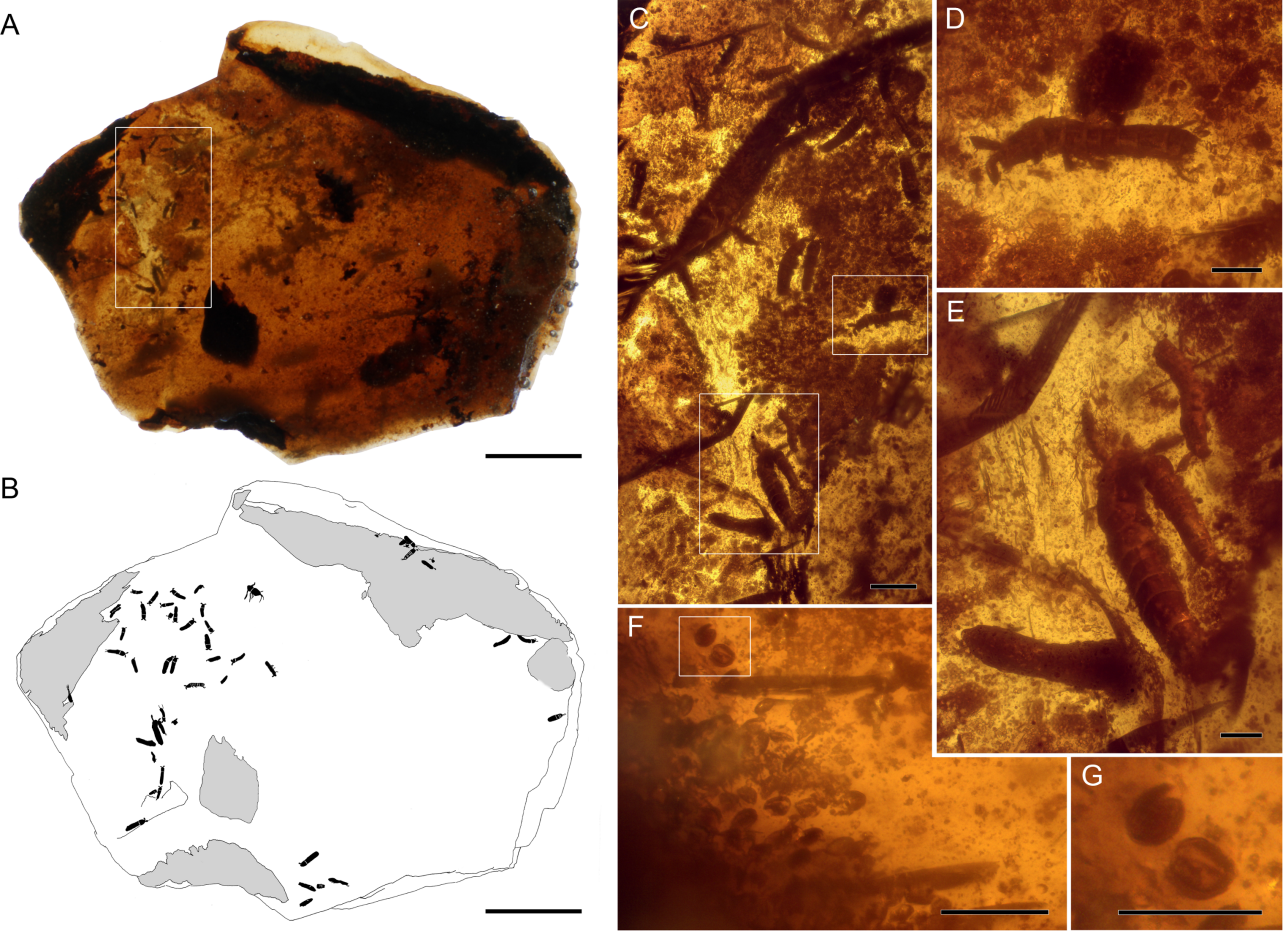
Assemblage evidencing gregariousness of *Proisotoma communis* in Early Cretaceous Spanish amber. (A) Overview of the amber piece MCNA 11231. (B) Camera lucida drawing showing the general configuration of the syninclusions. (C) Close up of box in (A), showing some individuals from the main group of the association. (D and E) Close up of boxes in (C), showing the intraspecific character variability described and mainly concerning the size of the specimens (see also S1 Table). (F and G) *Cycadopites*-type pollen grains from the assemblage (box in (F) indicate the magnified cluster in (G)), and possibly attributable to falling pollen being concentrated in the viscous resin. Scale bars, 2000 μm (A, B), 300 μm (C), 100 μm (D–F), 50 μm (G).

The new discovery consists of an aggregation of up to 45 specimens of *Proisotoma communis* in a small piece of Albian-aged amber from the Peñacerrada I outcrop (Fig 3 and S1 Text). The size distribution of individuals within the aggregation of *P*. *communis* shows a large range of sizes with a preponderance toward the smallest size classes (S1 Table). However, no differences concerning chaetotaxy, body proportions, antennae or furcular structure were observed. The impossibility to distinguish their genital openings, and the absence of secondary sexual characters precludes any gender assignment. Otherwise, differences in size reported in *P*. *communis* most probably correspond to intraspecific variation, because of overlapping consecutive developmental instars. Extant juvenile Collembola generally resemble adults except for the absence of mature sexual structures and some aspects of setal coverings. Juvenile springtails begin feeding soon after emergence from the egg. Growth is rapid as they pass through a number of instars and before attaining adulthood, and like other Entognatha they can molt multiple times after reaching sexual maturity [3]. We note that the presence of some cleared specimens in the fossil association are likely to be the exuviae of molts from a few individuals, just as occurs in modern aggregative assemblages [40]. This, along with the presence of particulate debris, coprolites, fungal hyphae, and plant remains (e.g. pollen, Fig 3F to 3G), suggest that the piece of resin in which the springtail association is embedded fell onto moist litter. This also appears to have been the case for those other Spanish amber pieces preserving springtails, and which also contain an assorted arthropod fauna typical of the forest litter [11]. Indeed, litter-inhabiting arthropods are relatively frequent in other pieces of amber from the same outcrop [45], suggesting that the resin flows occurred very close to or directly onto the ancient soil.

Many associations in amber are the result of random movements of organisms as they try to escape or are displaced after death by resin flows (‘dying’ or ‘death assemblages’), which may bring inclusions into close proximity with one another. However, those associations containing many individuals of the same taxon usually are not random [46, 47]. The present aggregation is likely to be the result of a behavioral phenomenon rather than an artifact of fossilization owing to the following: 1. Extant springtails often occur in groups, and sometimes form large aggregations, and so it is not unreasonable to expect a monospecific association such as that observed in the fossil; 2. Springtails in amber are often preserved in association with other springtails (e.g., samples MCNA 9273, MCNA 9612 and MCNA 10040, also preserved three, seven, and up to 20 individuals of *P*. *communis*, respectively); 3. Different developmental stages of the same species and exuviae are present; 4. The taphonomic analysis of the amber piece proved that it is made by a unique resin flow and such that the distribution of the springtails reflects a synchronous event; 5. The lack of significant body decay (Fig 3C to 3E) is indicative of rapid demise and little post-mortem movement within the resin; 6. All the springtails are well-preserved in a natural position and adjacent each other (Fig 3E), indicating that they were rapidly preserved after being trapped on the resin, while in a death assemblage (necrocenosys) they would be variously positioned in the amber; and 7. Given the skittish behavior of living springtails and their ability to leap from danger by release of the furca on the underside of the abdomen, the present preservation is indicative of extremely rapid entombment and almost instantaneous demise.

**Fig 4:**
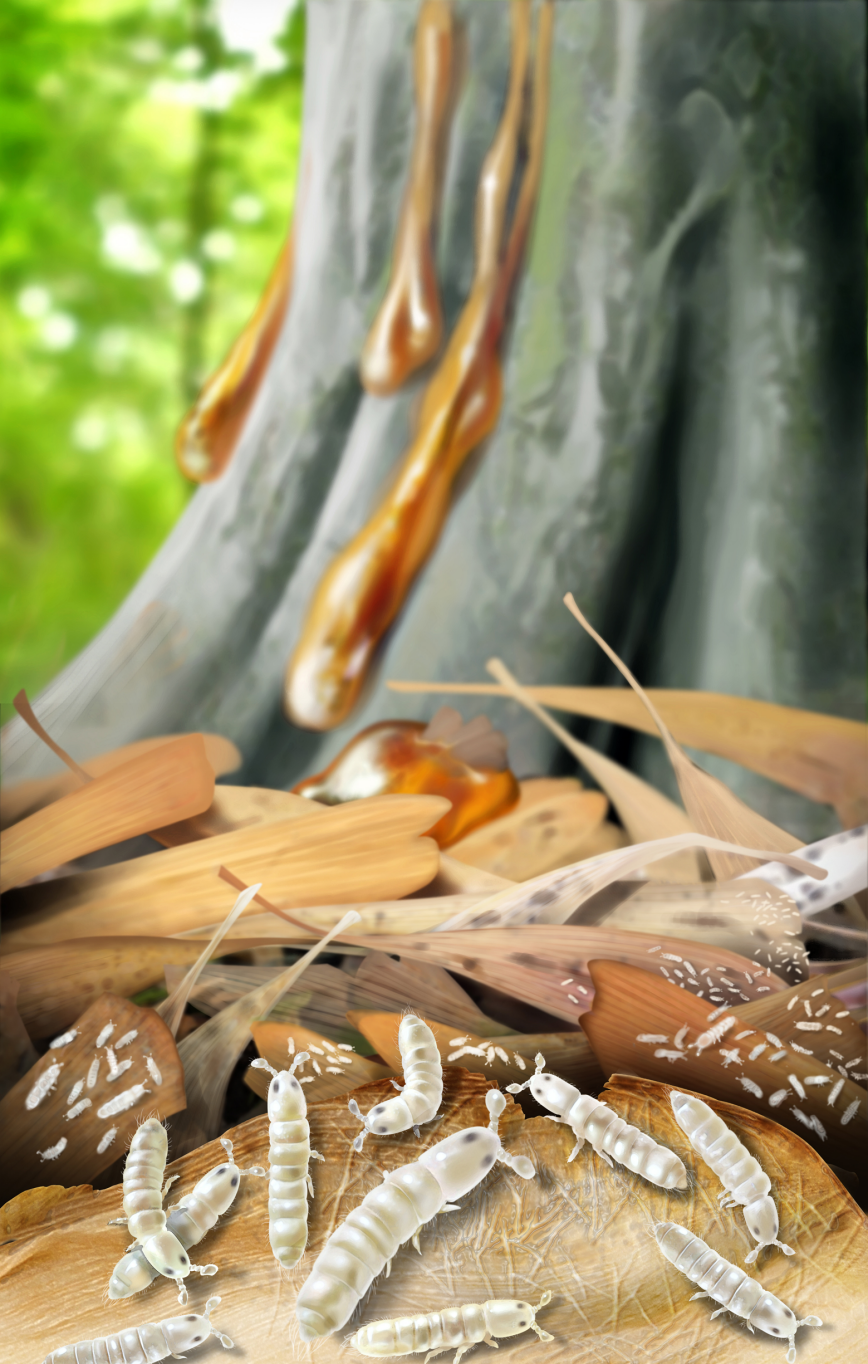
Reconstruction of an aggregation of *P*. *communis*. Leaves constituting the litter are of *Eretmophyllum* (Ginkgoales), a common plant macroremain as cuticles in the Spanish amber-bearing strata. Body color of the collembolans is conjectural but based on the coloration seen in the close extant relatives (artist J. A. Peñas, with scientific supervision).

Aggregation behavior in Collembola is not completely understood, nor the external cues involved in causing synchronized ‘colonies’. Even in uniform soils, springtails are rarely randomly distributed, but tend to be clumped or aggregated because of pheromones, ecological factors, or simply as a result of their biological activities (related with molting cycles and feeding phases). Collembola reproduce rapidly, and massive episodes of synchronised reproduction can result in huge numbers of immature forms [37]. This behavior is involved in migration and even allows species to cross habitats that would otherwise represent physical barriers in the landscape [43]. Aggregation is one of the most basic and widespread behaviors in arthropods and frequently has a social origin [48]. Information on aggregative strategies in Collembola is sparse and descriptive, but the discovery of an aggregation of *P*. *communis* (Fig 4) implies that the same pre-social behaviors were already well established by the Early Cretaceous, and likely originated much earlier in the history of Collembola. The fossil also sheds light on the complex litter communities of Early Cretaceous ecosystems, a habitat otherwise only known from fossil resins and scarcely recorded.

## Discussion

The remarkable inclusions reported here showcase the potential that the amber record offers to reconstruct not only the morphology of fossil arthropods but also their ancient life history, behaviors, and broader ecology and habitat. Given that in springtails entire suites of morphological characters are strongly correlated and influenced by their ecology [49], the species reported here and elsewhere [10, 11] permit us to infer the local environment in which the resin was exuded. Although pieces of amber from Peñacerrada I largely represent a sampling of taxa from above the forest floor, fossiliferous resins are just as likely to sample soil and litter faunas and even nearby aquatic habitats [50], microenvironments in which modern Collembola are abundant and diverse. In fact, there are various other arthropods preserved in the Spanish amber deposits that are indicators of a litter-dwelling to semi-aquatic fauna [45, 51], and that the general environment was likely near water or perhaps even representative of a swamp.

It is clear that the characteristic behaviors of the springtails discussed have persisted unchanged for a considerable interval of time, and that during this period the species have retained morphologies consistent with their unique life history. The stasis of these behaviors over such extended expanses of geological time reflects a general ethological bradytely among two lineages of Collembola and suggests that such antiquity might be more widespread across the class. Rather than being greatly labile, at least some behavioral repertoires in basal hexapods may conversely be tightly conserved. Ethological bradytely has also been documented from body and trace fossils among select insect groups [52–54]. It is becoming clear that highly specialized repertoires and ecologies, along with their associated morphological modifications, appeared early among select basal hexapods and were already established by the Early Cretaceous, and likely well prior to this. How expansively this may be generalized across Entognatha or the early diverging clades of insects, such as Archaeognatha and Zygentoma, remains a lingering question for the paleoethological and paleoecological investigation of Hexapoda.

## Supporting information

S1 Text Systematic Paleontology. (DOCX)

S1 Fig Fossil record of collembola. The numbered records of behavioral interactions are as follows: 1, phoresis; 2, spermatophores (indirect sperm transfer); 3, egg laying (stress behavior); 4, courtship; 5, aggregative behavior. Lebanese and French ambers denoted by an asterisk (*) have unstudied collembolan faunas. Note: †Protentomobryidae are assuredly a synonym of Isotomidae. For a detailed checklist of occurrences, refer to the catalog in [11].
(TIF)

S1 Table Measurements in microns of the specimens of *Proisotoma communis* from the assemblage. In bold are the maximum and minimum measurements.
(DOCX)
