# Supplementary material for: Mating and aggregative behaviors among basal hexapods in the Early Cretaceous
Source: PLoS One. 2018 Feb 21;13(2):e0191669. doi: 10.1371/journal.pone.0191669 (PMC5821437; doi:10.1371/journal.pone.0191669)
Supplement: S1 Text — (DOCX) [file pone.0191669.s001.docx]

**Supporting information**

**Systematic Paleontology**

This work, combined with the recent systematic studies of Early Cretaceous Collembola in Spanish amber, enables us to take a broader perspective concerning this diverse collembolan paleofauna. A total of over 102 specimens were identified, with those of the family Isotomidae (Entomobryomorpha) dominant and representing over 90% of the entire Spanish collembolan paleofauna. Symphypleonan appeared less abundant but diverse, and included at least three families (Sminthurididae, Katiannidae, and Sminthuridae: subfamilies Sphyrothecinae and Sminthurinae?), and the first Mesozoic records of Sminthurididae and Sphyrothecinae [10, 11]. Detailed systematic descriptions of the species have been previously published; herein we only briefly mention the two species from which behavioral information can be either directly or indirectly obtained.

Class Collembola Lubbock.

Order Symphypleona Börner; Family Sminthurididae Börner; Genus *Pseudosminthurides* Sánchez-García and Engel; *Pseudosminthurides stoechus* Sánchez-García and Engel.

*Comments.* The holotype male of *Pseudosminthurides stoechus* (Fig 2A) is preserved in a clear-yellow turbid piece of amber (MCNA 12788) trimmed to 1.0 × 0.9 × 0.1 cm (set into an epoxy resin trapezoid of dimensions 2.2 × 1.5 × 0.2 cm), and accompanied by abundant debris and arthropod remains (e.g., scales). The specimen is virtually complete but an internal fracture in the amber runs along the head and body obscuring some details, and the hind legs are obscured by the opaque body mass. The right antenna is lost, and the left antenna lacks the third and fourth articles (this feature not properly described in the original description [10]). As the second and third articles are those involved in the clasping function, it is plausible that they broke at the level of their articulation. Syninclusions include six acari, the holotype of *Protoisotoma* *autrigoniensis* Sánchez-García and Engel, and an additional entomobryomorphan springtail (*P.* *autrigoniensis*?).

Order Entomobryomorpha Börner; Family Isotomidae Schäffer; Genus *Proisotoma* Tullberg; *Proisotoma communis* Sánchez-García and Engel.

*Comments.* The amber piece MCNA 11231 (Fig 3) is trimmed to 1.2 × 0.9 × 0.3 cm (set into an epoxy resin trapezoid of dimensions 2.1 × 1.4 × 0.3 cm) and contains an association of up to 45 specimens of *P. communis* (among which 42 are virtually complete, and three are nearly complete), preserved together with disarticulated remains of several further springtails of the same morphotype (at least three disembodied heads, one furcula, and two partially preserved abdomens), and one Symphypleona (genus and species indeterminate). The thick and darkly colored amber piece is quite turbid (possibly as a result of its contact with litter), and the association of springtails is accompanied by much debris, coprolites, fungal hyphae, and plant remains (e.g., pollen), as equally occur for many amber pieces preserving Collembola. The accumulation of numerous *Cycadopites*-type pollen grains adjacent to some of the springtails in the association is noteworthy, and possibly attributable to falling pollen being concentrated in the viscous resin. Analysis with UV-light confirmed the absence of distinct layers composing the amber piece, and therefore proved that it is made by a unique resin flow and such that the distribution of the inclusions reflects a synchronous event.

The association of *P. communis* consists in a main group of specimens and a few more distributed along the margins of the amber piece. The single symphypleonan stands aside from the main group of *P. communis* (see Fig 3A and 3B) for a general view of the assemblage of syninclusions). Differences of density observed between specimens likely represent taphonomic disparities. This may be related to differences in the preservation of the cuticle and the inner organic structures, and differences between the hexapods before their entombment in the fresh resin, e.g., dead and/or moults versus living animals. Intraspecific character variability was low, and mainly concerns the size of the specimens. Body sizes range from 186 to 597 µm (Fig 3D and 3E and S1 Table) and the distribution of sizes shows that the smallest size classes contain the most individuals and the largest size classes the least. The genital openings, of little-use in collembolan taxonomy, remain obscured, and no other secondary sexual characteristics have been observed, thereby preventing gender assignment.
