# Supplementary figures and images for: Mating and aggregative behaviors among basal hexapods in the Early Cretaceous
Source: PLoS One. 2018 Feb 21;13(2):e0191669. doi: 10.1371/journal.pone.0191669 (PMC5821437; doi:10.1371/journal.pone.0191669)

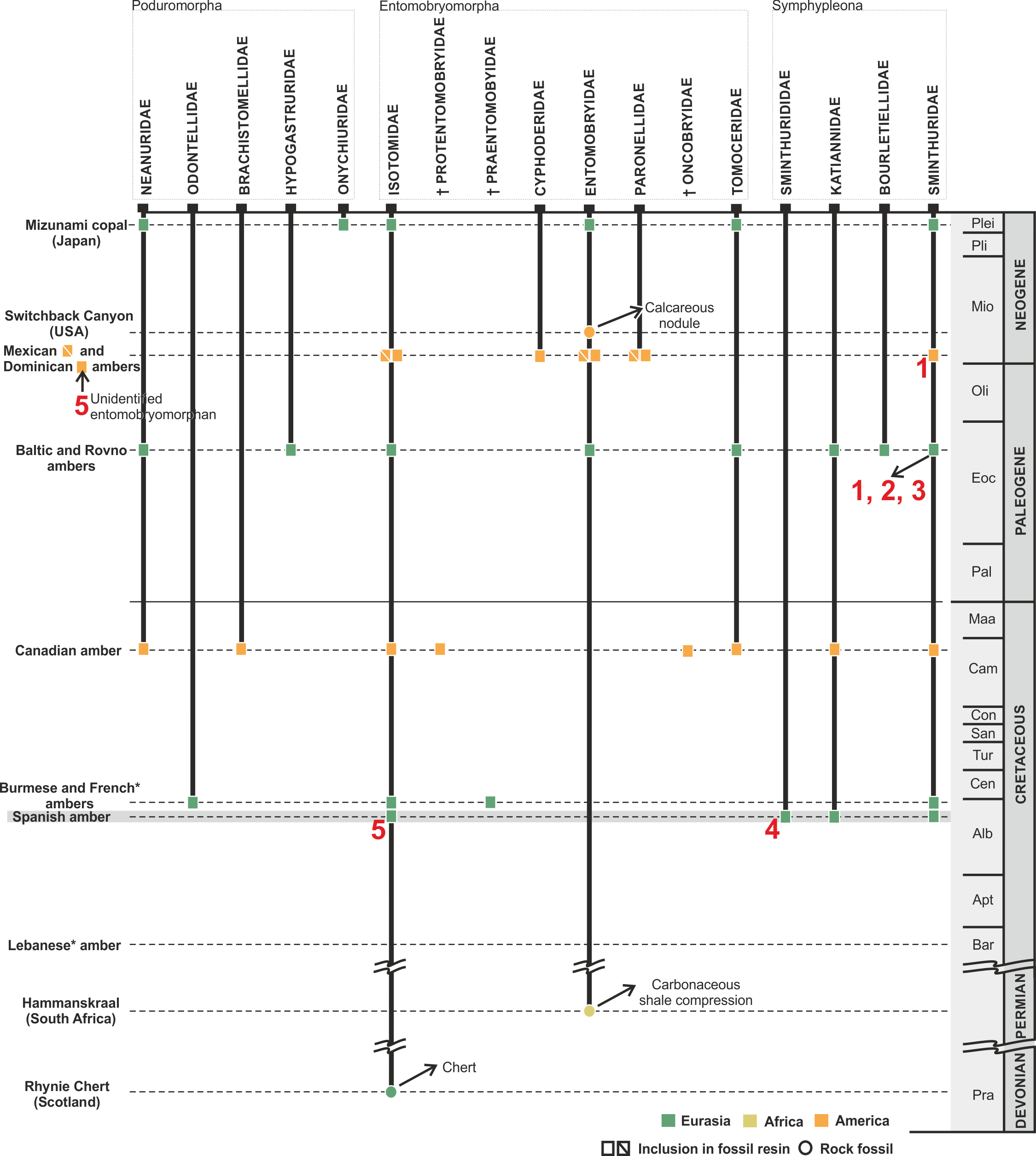

Supplement: S1 Fig — The numbered records of behavioral interactions are as follows: 1, phoresis; 2, spermatophores (indirect sperm transfer); 3, egg laying (stress behavior); 4, courtship; 5, aggregative behavior. Lebanese and French ambers denoted by an asterisk (*) have unstudied collembolan faunas. Note: †Protentomobryidae are assuredly a synonym of Isotomidae. For a detailed checklist of occurrences, refer to the catalog in [11]. (TIF) [file pone.0191669.s002.tif]
