# Supplementary material for: Mating and aggregative behaviors among basal hexapods in the Early Cretaceous
Source: PLoS One. 2018 Feb 21;13(2):e0191669. doi: 10.1371/journal.pone.0191669 (PMC5821437; doi:10.1371/journal.pone.0191669)
Supplement: S1 Table — In bold are the maximum and minimum measurements. (DOCX) [file pone.0191669.s003.docx]

**S1 Table.** **Measurements in microns of the specimens of *Proisotoma communis* from the assemblage.** In bold are the maximum and minimum measurements.

| Number | Body length (µm) | Number | Body length (µm) |
| --- | --- | --- | --- |
| 1 | 232.00 | 22 | 457.46 |
| 2 | 334.80 | 23 | 222.10 |
| 3 | 331.49 | 24 | 377.90 |
| 4 | 275.14 | 25 | 275.14 |
| 5 | 358.01 | **26** | **185.63** |
| 6 | 357.35 | 27 | 457.46 |
| 7 | 361.33 | 28 | 291.71 |
| 8 | 235.36 | 29 | 321.55 |
| 9 | 278.45 | 30 | 338.12 |
| 10 | 295.03 | 31 | 374.58 |
| 11 | 332.15 | 32 | 245.60 |
| 12 | 253.26 | 33 | 308.29 |
| 13 | 285.08 | 34 | 311.60 |
| 14 | 383.20 | 35 | 397.79 |
| 15 | 351.38 | 36 | 389.90 |
| 16 | 391.16 | 37 | 417.10 |
| 17 | 364.64 | 38 | 390.08 |
| 18 | 391.16 | 39 | 384.53 |
| **19** | **597.35** | 40 | 348.07 |
| 20 | 312.93 | 41 | 325.30 |
| 21 | 308.29 | 42 | 346.80 |
